# Recent Advances in Intracellular and *In Vivo* ROS Sensing: Focus on Nanoparticle and Nanotube Applications

**DOI:** 10.3390/ijms130910660

**Published:** 2012-08-24

**Authors:** Larissa M. Uusitalo, Nadine Hempel

**Affiliations:** Nanobioscience Constellation, College of Nanoscale Sciences & Engineering, University at Albany, SUNY, 257 Fuller Rd., Albany, NY 12203, USA; E-Mail: luusitalo@albany.edu

**Keywords:** nanoparticles, nanotubes, reactive oxygen species, hydrogen peroxide, ROS sensors

## Abstract

Reactive oxygen species (ROS) are increasingly being implicated in the regulation of cellular signaling cascades. Intracellular ROS fluxes are associated with cellular function ranging from proliferation to cell death. Moreover, the importance of subtle, spatio-temporal shifts in ROS during localized cellular signaling events is being realized. Understanding the biochemical nature of the ROS involved will enhance our knowledge of redox-signaling. An ideal intracellular sensor should therefore resolve real-time, localized ROS changes, be highly sensitive to physiologically relevant shifts in ROS and provide specificity towards a particular molecule. For *in vivo* applications issues such as bioavailability of the probe, tissue penetrance of the signal and signal-to-noise ratio also need to be considered. In the past researchers have heavily relied on the use of ROS-sensitive fluorescent probes and, more recently, genetically engineered ROS sensors. However, there is a great need to improve on current methods to address the above issues. Recently, the field of molecular sensing and imaging has begun to take advantage of the unique physico-chemical properties of nanoparticles and nanotubes. Here we discuss the recent advances in the use of these nanostructures as alternative platforms for ROS sensing, with particular emphasis on intracellular and *in vivo* ROS detection and quantification.

## 1. Introduction

The role of reactive oxygen species (ROS) during cellular signaling is ever expanding. Intracellular, sub-lethal changes in ROS primarily influence cellular signaling by thiol oxidation within target proteins, resulting in alterations in their structure-function properties [1]. Oxidation has been attributed to activating kinases and inhibiting phosphatases, leading to an overall enhancement of phosphorylation cascades [2,3]. In addition, conformational changes as a consequence of oxidation may lead to changes in protein stability, protein-protein and protein-DNA interactions and subcellular localization [4,5]. Redox regulation has been attributed to signaling cascades such as the HIf-1, NFκB, PI3K and MAPK pathways and shown to contribute to a diverse range of cellular responses, including regulation of transcription, proliferation, migration, metabolism, survival and inflammatory response.

Effective redox signaling is dependent on the balance between spatio-temporal ROS production and efficient scavenging by cellular antioxidants. The major intracellular ROS sources are NADPH oxidases (Nox) and the mitochondria. ROS emanating from the mitochondrial electron transport chain are slowly being linked to regulating cellular signaling [6–9]. However, a plethora of signaling pathways have been implicated to involve Nox family members, which produce superoxide (O_2_^−^) as a consequence of exogenous and endogenous cellular stimuli [8–10]. O_2_^−^ is rapidly converted to hydrogen peroxide (H_2_O_2_) by superoxide dismutases (Sod). Aberrant redox signaling, due to altered ROS production or changes in the antioxidant arsenal (including Sod, catalase, glutathione peroxidase, thioredoxin, peroxiredoxin and the glutathione pool), has been observed in a number of pathophysiological conditions including cancer and inflammatory disease.

## 2. ROS Sensors

Given the ever expanding role of sub-lethal intracellular ROS fluxes during cellular signaling it is imperative to develop live cell and *in vivo* sensors that are able to monitor subtle, spatio-temporal ROS fluxes in real time. Preferably, these sensors should also be able to discern the biochemical nature of the ROS in question. In the past, researchers have largely relied on the use of dyes for ROS sensing, due to their sensitivity, high signal-to-noise ratio, cell permeability and ease of measurement. Examples of the most commonly used probes used are the oxidation products of hydroethidium (HE), used for O_2_·^−^, and dichlorodihydrofluorescein diacetate (DCFH-DA), used for H_2_O_2_ sensing. The specificity and accuracy of these dyes have been in scrutiny for some time. In particular, H_2_O_2_ does not directly oxidize DCFH-DA, has been shown to lack specificity for a particular ROS, can directly result in the creation of further ROS and thiol oxidation, and shown to interact with cytochrome c, rather than ROS as a consequence of apoptosis [11–17]. Research is underway to develop specific and highly sensitive fluorescent ROS probes to circumvent these issues (e.g., boronate-based probes for H_2_O_2_) [18–22]. As the topic is beyond the scope of the present manuscript we refer the reader to the following review articles addressing the caveats associated with DCFH-DA ROS sensing [23,24]. In addition, to improve *in vivo* imaging with fluorescence probes, there is a need to eliminate high background from tissue auto-fluorescence and improve tissue penetrance of the signal. This can be achieved by pushing the excitation and emission spectrum to the near infra-red [25,26].

The inherent instability and irreversible nature of some fluorescent probes also make long-term imaging problematic. This has led the field to develop genetically encoded fluorescent proteins, such as RxYFP, RoGFP and HyPer, to enable transient live-cell imaging [27–31]. These proteins are engineered to alter their fluorescence spectrum upon oxidation and therefore allow for ratiometric quantification without the need for a reference dye. They are also reversible, dependent on the intracellular thiol and antioxidant enzyme pool. Recombinant fluorescent proteins can be sub-cellularly targeted, but require adequate cellular expression and do not lend themselves to *in vivo* studies unless expressed by a transgenic animal.

Evidence suggests that different reactive oxygen and nitrogen species (RNS) may have very different roles in affecting cellular signaling. For example, H_2_O_2_ displays a number of features that make it a prominent candidate as a second messenger in ROS-driven signaling events. Compared to other ROS, such as O_2_·^−^ and ·OH, H_2_O_2_ is relatively stable and able to readily traverse cellular membranes [4]. Given an important role for H_2_O_2_ as a potential second messenger, it is imperative that we develop molecular probes to accurately measure small fluxes in this particular ROS, without erroneous contribution by other ROS or RNS. Efforts are underway to improve the specificity of sensors towards individual ROS species, which will aid our understanding of the complex contributions different ROS species may play in cellular signaling.

It is evident that ROS fluxes involved in cellular signaling appear in specific cellular locations. Intracellular ROS sensing must therefore be highly sensitive, specific and able to be resolved in a spatio-temporal manner. Furthermore, *in vivo* applications demand that the probe be stable, preferably reversible and, importantly, non-toxic. Below we will highlight recent advances in the use of novel Nanoparticle (NP) and carbon nanotube (CNT) application for intracellular and *in vivo* ROS sensing, which aim to address some of the caveats associated with traditional methods (Table 1).

## 3. Nanoparticle-based Sensors

There has been exponential use of NPs in the field of sensing due to their unique physico-chemical properties. Although the term “nanoparticle” can be associated with an infinite number of different particles, NPs are generally characterized as having dimensions below 100 nm in diameter, with a high surface-to-volume ratio. While aspects of specific NPs used for ROS sensing will be discussed below, there are a number of general advantages to the use of NPs in cellular and *in vivo* sensing and imaging. These include the relative stability and ease of delivery of particles into cellular systems. The chemical make-up of NPs is easily manipulated and altering the surface chemistry through conjugation with different molecules allows for enhanced targeting to cells and subcellular compartments. Interestingly, NPs have been exploited for *in vivo* targeting and imaging of tumor tissues. Even without specific conjugation of targeting molecules, their size enables NPs to be taken-up and retained by tumors, an effect referred to as enhanced permeability and retention, which is a characteristic of the tumor and its vasculature [32,33]. Surface conjugation of other molecules, such as fluorescent dyes, proteins or DNA, provide infinite possibilities in NP design for specific functions. Below, we will categorize ROS sensing NP applications based on their physical properties, namely non-metallic and metal-based NPs.

Another attractive property of NPs is their ability to be “loaded” with a vast variety of molecules, making NPs attractive carriers and physical protectors of the encapsulated/conjugated cargo. Examples of these include micelle-like NPs and the matrices of polymeric NPs. While dyes usually rely on passive uptake by cells, NP loading allows for more targeted delivery of the probe. In addition, a reference dye can be loaded into the same particle allowing for equal access of the reference dye to the cell and for quantitative ratiometric analysis of the sensor dye. Encapsulation or embedding can be made more complex by designing multiple cores within the NP matrix. This is advantageous when, for example, a reference dye is loaded in the inner core and a sensor dye in the outer matrix, keeping the reference dye unaffected by the analyte. This loading also prevents degradation of the sensor or molecule by the cell and inhibits non-specific binding of the encapsulated molecule with cellular components. Moreover, some NPs have intrinsic optical and physical properties that allow for detection of analytes without the use of fluorescent dyes, such as gold-NPs (Au-NPs) and quantum dots (QDs) [69]. Optical Nanosensors are also often referred to as Photonic Explorer for Bioanalysis with Biologically Localized Embedding (PEBBLE) Nanoparticles [70,71] and are often categorized into two types. Type 1 optical NPs contain a senor that detects the analyte and also transduces the signal, similar to traditional dyes. Type 2 NPs contain a separate analyte detector and transducer and often rely on manipulation of the intrinsic optical properties of the NP itself upon analyte binding to the detector portion. For a summary of recent NP-based ROS sensor applications we refer the reader to Table 1.

### 3.1. Non-Metallic NPs

Non-metallic, solid NPs used for ROS sensing are generally polymer-based, allowing for encapsulation of the detector and signal transducer components, such as ROS-sensitive dyes.

#### 3.1.1. NPs Embedded with ROS-Sensitive Fluorescence Dyes

**Figure 1:**
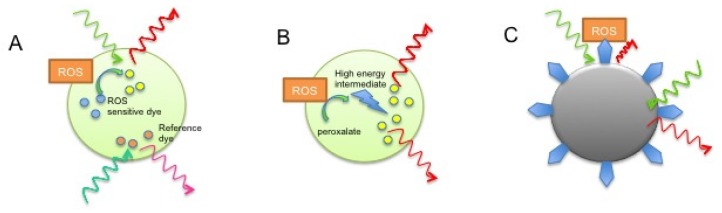
Examples of Nanoparticles (NPs) adapted for ROS sensing (**A**) Polymer-based NPs embedded with ROS-sensing and reference fluorescent dyes; (**B**) Chemiluminescent NPs; (**C**) Metallic NP fluorescence quenching upon oxidation of functionalized ROS sensitive molecules (blue).

As mentioned above, fluorescence sensors have the advantage of providing a high signal-to-noise ratio with high sensitivity and relative ease of detection. Embedding ROS-sensing dyes in polymeric NPs provide advantages, such as inhibiting interaction of the dye with intracellular proteins, protecting the dye somewhat from degradation and inhibiting undesired sequestration into subcellular compartments. In addition, loading of a reference dye allows for accurate ratiometric calculations of the ROS signal (Figure 1A). Kim *et al*. recently reasoned that specificity of DCFH-DA for H_2_O_2_ can be achieved by encapsulating the dye in organically modified silicate (ORMOSIL) NPs [34]. The authors describe cellular targeting of NPs to macrophages using a TAT-peptide to enhance membrane penetration and potentially prevent phagocytosis, as the probe is pH sensitive, a common feature of ROS-sensing probes. With this probe the authors reported sensing of low nM H_2_O_2_ levels and intracellular H_2_O_2_ bursts following macrophage stimulation. The authors argue that short-lived ROS such as ·OH cannot penetrate into the center of the NP due to time constraints, and that other ROS and RNS are excluded due to the hydrophobic energy barrier of the NP. Furthermore, size exclusion prevents access of alkylperoxyl radical and proteins such as esterase and HRP to the dye. The problem that remains with this proposed concept is the fact that H_2_O_2_ does not directly interact with DCFH-DA, but rather its hydrophilic derivative, which requires esterases for DCFH-DA hydrolysis. Since esterases presumably will not have access to the NP matrix this will present a fundamental problem to use of this NP in H_2_O_2_/ROS sensing. While the authors argue that DCFH-DA fluorescence can directly be induced by H_2_O_2_ in their system this is generally not considered to be an accurate assumption and questions the validity of their NP design [34]. ORMOSIL NPs have also been used to sense singlet oxygen using 9,10-dimethyl anthracene, again providing improved selectivity due to the hydrophobic nature of the matrix, inhibiting access to short lived and polar ROS [35]. Variations of NPs containing ROS-sensitive fluorescent dyes have also been used to detect other intracellular ROS and RNS, such as peroxinitrite and ·OH, and often contain a reference dye embedded in the matrix for ratiometric quantification [36–38]. An alternative to direct interaction of ROS with the sensor dye has been explored by encapsulating horseradish peroxidase (HRP) into NPs. In one study, H_2_O_2_ was used as a substrate by HRP to oxidize the target dye Amplex Red and shown to sense exogenously applied H_2_O_2_ and LPS induced ROS changes within macrophage cells [39].

While the above fluorescent dyes are highly sensitive, their short excitation and emission wavelengths do not allow for effective *in vivo* sensing. This is primarily due to high auto-fluorescence of tissue components, such as collagen, and low penetrance of dye-associated wavelengths, as a consequence of absorption by biological molecules, such as heme-containing proteins [25]. Use of fluorescent probes in the near-infrared region (600–1000 nm) circumvents these problems. For example, hydrocyanine-conjugated chitosan functionalized pluronic-based NPs were able to sense inflammatory ROS surges in murine tumor xenografts. Chitosan-functionalized NPs were previously shown by the investigators to have intrinsic tumor targeting. ROS sensing was achieved by monitoring fluorescence recovery after oxidation of hydrocyanine to cyanine (Cy5.5) at 693 nm [40]. The probe however lacked specificity (responsive to H_2_O_2_, O_2_·^−^, ·OH, KO_2_) and sensitivity to sub-μM levels of ROS tested *in vitro*, and may thus be only applicable to large ROS-surges in response to inflammation. Great care should be taken in interpreting specificity data from NP-ROS-sensing dye conjugates. Simple encapsulation does not circumvent the caveats associated with these dyes (as described above), such as specificity. Efforts are under way to develop ROS-species specific near-infrared dyes, such as the boronate-cage based fluorescent dye naphtha-peroxyfluor-1 [72], which may be promising novel strategies for combination with NP intracellular delivery.

#### 3.1.2. Luminscent NPs

Luminescence has greatly been exploited for many *in vivo* imaging applications, as it does not require an optical excitation signal and often relies on near-infrared emission wavelengths to improve deep tissue imaging [73]. Chemiluminescent NPs have been successfully used to image H_2_O_2_ *in vivo*. In one study, polymeric peroxalate NPs were loaded with a pentacene fluorescent dye, where H_2_O_2_ reacts with the peroxalate ester to form a high-energy intermediate, dioxetanedione (Figure 1B) [41]. In turn, this excites the fluorophore, which emits in the near-infrared range (630 nm). Following intraperitoneal (I.P.) injection of these NPs, the investigators were able to sense H_2_O_2_ in an LPS-induced murine inflammation model [41]. These NPs however do not lend themselves to intravenous (I.V.) injection because of their large size (500 nm) and enhanced clearance due to their hydrophobic surface. In subsequent reports the authors improved on this principle by encapsulated the fluorophore in PEG-peroxalate ester containing polynorborne micelles [42], as well as changing the chemiluminescent agent to poly(ethylene glycol)-*b*-poly(e-caprolactone) [43]. These NPs had reduced size, enhanced circulating life and were able to efficiently detect H_2_O_2_ at physiologically relevant concentrations (50 nM). Lim *et al*. similarly constructed a chemiluminescent pluronic NP, which contained the reactive bis[3,4,6-trichloro-2-(pentyloxycarbonyl)phenyl] oxalate that upon oxidation was able to induce Cy5 chemiluminescence encapsulated within the NP. It was shown that this probe could similarly detect LPS-induced inflammation in a murine model and the authors were able to use the NP to indirectly measure glucose levels in their system [44]. Recently, these NPs were further adapted for photodynamic therapy, whereby H_2_O_2_ induction of the chemiluminescent signal activates the Photodynamic therapy agent mesotetraphenylporphine, aiding killing of C6 and LoVo cell lines treated with sub-lethal levels of 0.2 μM H_2_O_2_ *in vitro* [45]. Increases in intracellular ROS have been observed in a number of tumor cells. It remains to be determined if intrinsic increases in cellular ROS are able to activate this NP encapsulated photodynamic agent. While these studies are only in their infancy, it is attractive to consider that, together with tumor targeting, the altered intracellular ROS environment of tumor cells may provide an additional tumor-specific therapy-activation step that could be considered for future NP-drug delivery development. Tumor-specific activation or drug release from NPs has already been investigated using pH sensitivity and tumor specific protease activation. For example, ovarian cancer xenografts were successfully targeted following release of paclitaxel from pH-sensitive NPs, once taken up into the hypoxic and high pH environment of the ovarian cancer cells [74].

### 3.2. Metallic NPs

Metallic NPs, such as gold (Au) NPs and quantum dots (QD) have intrinsic optical properties that can be exploited for sensor design. QDs are semiconducting NPs that display inherent fluorescent properties, which can be altered depending on particle size, make-up (e.g., CsTe, CsSe) and surface modification [69,75–77]. The advantage of these over traditional fluorescent dyes is a much stronger signal, lack of photo-bleaching and enhanced stability in solutions.

#### 3.2.1. Metallic NP Fluorescence Quenching

Taking advantage of the intrinsic optical properties of metallic NPs has aided in the development of a few novel strategies towards ROS sensing, most of which are yet to be trialed intracellularly or *in vivo*. Surface functionalization has been shown to significantly alter the fluorescent properties of metal NP and nanoclusters and changes in the structure of the functionalized molecules may further alter the fluorescence properties of the NPs [77,78]. For example, conjugation of horse radish peroxidase (HRP) to Au nanoclusters resulted in a characteristic emission spectrum, which was quenched in a dose dependent manner by H_2_O_2_ addition [46]. The NP was quenched with H_2_O_2_ concentrations as low as 100 nM, but was not ROS selective, as others, such as O_2_·^−^, had similar quenching effects [46]. The authors speculate that H_2_O_2_ induces a conformational change in HRP, which is reflected in the surface chemistry of the nanoclusters and hence a quenching of fluorescence. Since these NPs have an emission spectrum of 650 nm, close to the near-infrared spectrum, there is a potential that these probes have future applications in cell and *in vivo* sensing. More specific H_2_O_2_ sensing was achieved with 11-mercaptoundecanoic acid-bound (MAU) Au-NPs, where oxidation released the compound from the S-Au bond, effectively decreasing the strong fluorescent signal of the MAU-bound Au-NP [47]. An O_2_·^−^ selective sensor was created in a similar fashion using negatively-capped CdSe/ZnS QDs conjugated to oxidized Cytochrome *c* [48]. Here, pyrogallol-derived O_2_·^−^ readily reduced the oxidized Cytochrome *c*, leading to enhanced fluorescence, in a dose dependent manner (lowest detection 80 nM). Further, the investigators demonstrated that this probe was able to detect O_2_·^−^ changes following HeLa cell stimulation with PMA and showed low cellular toxicity compared to traditional ROS dyes. Altering quenching of QDs has also been exploited for NO sensing, for example with Tris (N-(dithiocarboxy)sarcosine)iron(III) linked QDs, where NO binding to the iron complex restores the fluorescent signal of the NP [49].

To detect temporal changes in intracellular ROS levels, Casanova *et al*. engineered europium Eu^3+^ doped NPs that work on the principle that Eu^3+^ can be photo-reduced to Eu^2+^, decreasing luminescence signal of the NP [50]. The re-oxidation to Eu^3+^ by ROS can thus be monitored by the recovery of luminescence. Interestingly, the investigators were able to sense intracellular ROS surges following PDGF stimulation of cells, which was blocked by Nox inhibition using apocynin treatment [50]. Given the reversibility and stable nature of this NP, effective targeting to subcellular locations will make this an attractive tool for spatio-temporal sensing of intracellular ROS.

#### 3.2.2. NP Surface Energy Transfer (NSET)

NP surface energy transfer (NSET) relies on the intrinsic property of Au-NP electrons to dampen a near-by or conjugated fluorophore’s oscillating dipole [76,79–82]. For example, these have been used successfully to detect subtle changes in length and sequence of fluorophore-conjugated oligonucleotides [80]. In addition, any mechanism that frees the fluorescent molecule from the Au-NP can hence enhance the fluorophore’s signal. This has also been exploited to monitor enzyme cleavage events [83]. It has also been considered a feasible alternative to Förster resonance energy transfer (FRET). NSET has been applied to intracellular ROS sensing, where Au-NPs were conjugated with fluorescein-hyaluronic acid (HA) and dopamine [51]. The dopamine was included to ensure intracellular stability against glutathione, and the probe was shown to be unaffected in highly reducing environments. While not specific, the probe was able to detect O_2_·^−^ and ·OH at low μM concentrations and showed enhanced fluorescence compared to DCFH-DA for these ROS species. This is based on the principle that ROS elicit degradation of high molecular weight HA, thereby releasing the fluorescent label from the vicinity of the Au-NP. Dose curves for H_2_O_2_ were not provided by the authors, but LPS-induced ROS surges in macrophages were readily detected using this probe and dampened by antioxidant application [51].

#### 3.2.3. Surface Enhanced Raman Scattering/Spectroscopy (SERS)

On traditional surfaces, Raman spectroscopy, which relies on the optical detection of changes in vibrational modes of molecular bonds, is thought to be of low sensitivity compared to fluorescent dyes. However, the physical properties of NPs enhance Raman scattering due to their enhanced plasmon resonance [84]. Any change in plasmon resonance following bond formation with specific molecules can be visualized optically by SERS in the near-infrared spectrum, making these NPs suitable for cellular and *in vivo* imaging. A special NP is needed, which usually consists of a silica core surrounded by a gold shell [84]. For ROS sensing these NPs are coated with molecules susceptible to oxidation, which changes their SERS spectrum. Auchinvole and colleagues used 1,8-diaza-4,5-ditihia-1,8-di(2-chloro-[1,4]-napthoquinone-3-yl)octane and 2-mercaptobenzene-1,4-diol conjugated-NPs, which are quinone-based molecules susceptible to reversible oxidation. Oxidation of these compounds induces a conformational change that is reflected in a shift in the NPs Raman spectrum [52]. Using this non-toxic, cytoplasmic-targeted SERS NP the investigators were able to sense intracellular redox potential in response to ROS inducers and following H_2_O_2_ exposure at levels able to induce apoptosis.

## 4. Nanotube-based Sensors

Carbon-based nanotubes (CNTs) represent a second class of nanoscale sensors showing promise in the detection and monitoring of H_2_O_2_ and other ROS. Using chemical vapor deposition, both single-walled CNTs (SWNTs) and multi-walled CNTs can be readily produced with minimal impurities [85–87]. An infinite variety of CNTs can be generated in this process, resulting in both metallic and semiconducting forms. The ability of semiconducting SWNT to fluoresce in the near-infrared region facilitates their use as biosensors both *in vivo* and *in vitro* [88,89]. As with QDs, SWNT biosensors are not susceptible to photo-bleaching common to molecular fluorophores, and are very stable in solutions. The hindered bioavailability and insolubility of SWNTs in aqueous environments is remedied typically by functionalization, either with covalently-bound molecules or non-covalent associations with larger macromolecules, such as DNA [56,90–93]. For a summary of recent CNT-based ROS sensor applications we refer the reader to Table 1.

### 4.1. Electrochemical CNT Sensors

To date, the vast majority of H_2_O_2_ biosensors developed using CNTs are electrochemical in nature. SWNTs promote electron transfer by enhancing the electrochemical activity of many biological molecules [57,58]. The conductivity of a CNT is highly sensitive to the presence of molecules adsorbed to its surface, a property that is highlighted by these sensors. Electrochemical enzyme biosensors are typically composed of a SWNT-coated electrode substrate to which an oxidase or dehydrogenase is covalently immobilized [57,59]. The CNTs are dispersed across the surface of the electrode within a thin film or in a vertical array, commonly referred to as a CNT forest. Nafion is ideally used instead of sulfuric acid solution films as it is more biocompatible and has greater electrical conductivity than traditional materials, making it an ideal substitute [90]. These arrays permit direct electron transfer between the enzyme’s functional groups and the electrode surface, eliminating the need for redox mediators and allowing for reagent-less sensor arrays. While many electrochemical sensors for H_2_O_2_ generation by fixed enzymes have been generated [53–60,94], those created by Yu and colleagues are among the most successful [55]. In construction of the sensors, two different enzymes, HRP and myoglobin, were separately bound to the tips of SWNTs that had been aligned vertically on a glassy carbon electrode. This relatively simple design boasted a small limit of detection and considerable sensitivity, with the best results coming from the HRP-immobilized sensor array. While we can gain useful information from these cell-free experiments regarding protein function and reactivity with electrochemical sensors, they have little utility in signal detection at extremely low concentrations and are currently not used within cells and tissues.

### 4.2. Optical CNT Sensors

**Figure 2:**
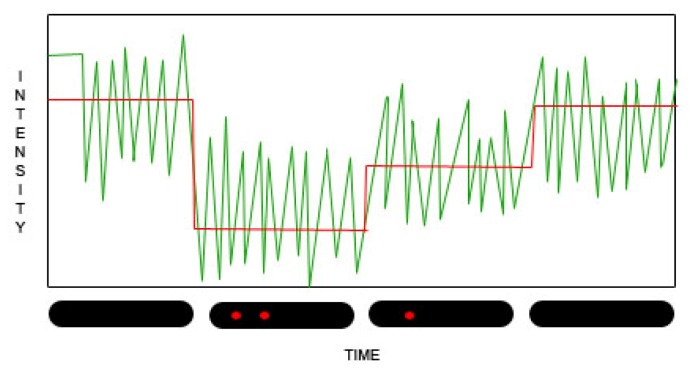
Stepwise quenching of nanotube fluorescence Fluorescent intensity measurements (gray) indicate the transitions between quenching states, as redox mediators partially draw away and release electrons back to the nanotube. These can be converted using a variety of algorithms into a stepwise representation of nanotube fluorescence dynamics (black) indicating the association with or dissociation of single molecules from the nanotube’s surface, as depicted below the graph.

The second class of SWNT H_2_O_2_ biosensors holds great promise for the advancing research in ROS and expanding knowledge about their involvement in cell signaling pathways. These sensors utilize the CNTs’ optical properties to permit detection at the single-molecule level. Chemical reactants at the nanotube surface can disturb the distribution of electrons within the nanotube, effectively protonating the sidewall of the nanotube and disturbing exciton-exciton recombination, which quenches fluorescence to some degree [63,95–97]. This quenching, measured as a decrease in the overall fluorescent signal of the nanotube, can be quantified using different algorithms to determine how many molecules are adsorbed to the surface of the nanotube and removing electrons, at any given moment [7,88,98]. This reaction, handily, is reversible. As the transient attraction between the nanotube and the reactant ends, the nanotube is deprotonated and normal fluorescence is reestablished. The quenching and dequenching reactions can occur many times over, as the CNTs are markedly stable (Figure 2).

Early work by Song *et al*. [64] illustrated the use of sodium dodecyl sulfate (SDS)-encased SWNTs as optical H_2_O_2_ sensors, which were able to detect H_2_O_2_ in the low μM range and shown to be reversible by the addition of catalase, H_2_O_2_ dialysis, and pH adjustments. This work was expanded upon with the generation of an optical glucose sensor [65]. Glucose oxidase (GO_x_) immobilized on SDS-SWNTs reacts with β-d-glucose to produce H_2_O_2_ and d-gluconic acid, both of which can be detected by the sensor. While immobilization of GO_x_ to SWNTs did not significantly alter SWNT emission spectra, there was a dose-dependent H_2_O_2_-driven suppression of the observed spectra following treatment with glucose. Karachevtsev *et al*. used GO_x_ immobilized onto DNA-SWNTs to produce a similar sensor [63]. Potassium ferricyanide (PFC) was used as a redox mediator, which quenched nanotube fluorescence in keeping with its ability to partially transfer an electron away from the nanotube surface. Upon H_2_O_2_ binding PFC was reduced, shifting electrons back to the nanotube and reestablishing fluorescence selectively. Other glucose sensors of similar design have been fabricated [66,99] and patented for commercial applications [67]. The above experiments, however, were conducted under cell-free conditions.

Pioneering the field of single-molecule detection at the cellular level, Strano and colleagues have used a SWNT/collagen film array tuned by roughness and porosity for H_2_O_2_ sensing in several applications [61,62]. Given that H_2_O_2_ is the only ROS stable enough and likely to diffuse from the cell to the SWNT/collagen film beneath it makes this method of detection relatively selective for this ROS. Jin *et al*. explored the origins of H_2_O_2_ signals produced by epithelial growth factor receptor (EGFR) using this single-molecule detection technique [61]. EGF binding to its receptor stimulates H_2_O_2_ production and regulates cell growth and proliferation. Both living and fixed A431 and 3T3 murine fibroblast cells were subjected to treatment with EGF while spread on SWNT/collagen matrices, and stepwise approximations of quenching events were generated from the observed signals using hidden Markov models. Localization of H_2_O_2_ production was achieved using Monte Carlo simulation of random global quenching scenarios. Simulation data were used to correct experimental data based on an observed probability density function in order to achieve localization of H_2_O_2_ production, which was ascertained to be at the membrane surface. The SWNT/collagen array has also been used to assess the role of H_2_O_2_ in the pro-angiogenic properties of europium (III) nanorods in endothelial cells (EC) [62]. As before, EC were cultured on the surface of the detection array and either the nanorods or VEGF, a known pro-angiogenic cytokine that stimulates the intracellular production of ROS, were applied. In order to relate concentration to fluorescence, the number of stepwise transitions generated over the observation period was determined by subjecting individual SWNT time-traces to a step-fitting algorithm and creating best-fit regressions that minimized error between the stepwise function and the fit curve of emission intensity. With this calibration in place, Strano’s group evaluated the amount of H_2_O_2_ generated on the surface of a human umbilical vein endothelial cell (HUVEC) in the presence of VEGF or europium(III) nanorods. Under stimulation with VEGF, H_2_O_2_ production at the HUVEC membrane’s outer surface increased 10-fold, whereas no increase was found with the nanorods, suggesting an alternate pathway was responsible for their pro-angiogenic effects. However, a second study using the SWNT/collagen array, showed that Eu^3+^ nanorods could induce ROS production within cells [100].

NO similarly has been detected on the single molecule level using SWNTs functionalized with a d(AT)_15_ oligonucleotide sequence that imparts the selectivity of the sensor [101]. Detection of molecular binding with the NT also occurs via a fluorescent signal [101]. Kim *et al.* used a similar sensor for intracellular and *in vivo* detection of NO [68]. Here, SWNT were “wrapped” in a 3,4-diaminophenyl-functionalized dextran to selectively sense NO, which bleaches the SWNT fluorescence at near-infrared. The reversible nature of this probe and imaging at these wavelengths allowed the investigators to trial it intracellularly and *in vivo*. LPS induced NO production could be measured by SWNT that had been taken up by macrophages and allowed for visualization of spatio-temporal changes in NO levels within cells. Further, addition of SWNTs in dialysis membranes into the abdomen of mice, allowed detection of signal changes following 60 μM NO administration. While ROS sensing by SWNT currently relies on the fixation of CNTs to a sensor array below the cell, these studies highlight the potential feasibility of using SWNTs for intracellular and *in vivo* imaging of ROS.

## 5. Conclusions

The field of NP and CNT sensors is ever expanding and improved methods for ROS sensing using these approaches will likely aid us in developing more specific, sensitive and reversible probes. Of vital importance to the continued study of ROS signaling is the ability to experimentally detect production of these signaling molecules intracellularly and *in vivo* at physiologically relevant concentrations. Unique properties of nanoscale probes that should be exploited for design of better ROS probes include:

- Stability: do not degrade easily in solutions.
- Intrinsic optical and electrochemical properties, can avoid the use of fluorophores.
- Optical properties in the near-infrared range, advantageous for whole animal imaging.
- Infinite possibilities for functionalization and conjugation with molecules.
- Easy cellular and sub cellular targeting.

While most probes described above sense intracellular ROS changes following stimulation with agents, such as PMA and LPS, which result in high ROS surges associated with immune response, the quest remains to develop ever sensitive probes that are able to detect localized subtle bursts of ROS within a cell. Currently, it is relatively difficult to accurately measure these subtle spatially localized changes in ROS, which are likely very important in the regulation of localized cellular signaling. For example, of interest to our own research would be to observe localized changes in ROS production at the leading edge of migrating cells in a 3D tumor environment. The unique properties of semi-conducting CNTs, which lend themselves to single molecule sensing, are a promising future method to explore.

Novel redox probes should also address the biochemical nature of the ROS to be sensed. As we are starting to unravel the role of specific reactive oxygen and nitrogen species involved, it is becoming apparent that they may have very divergent roles during cellular signaling, based on their stability, reactivity and ability to spatially localize to different regions within the cell. NPs and CNTs are readily taken up by cell lines and *in vivo via* passive uptake, although the described NPs will require further adaptation to improve bioavailability and effective cellular uptake by target tissues. In addition, given different sources of ROS within cells, design of future NP ROS probes should consider sub-cellular targeting, such as the plasma membrane (e.g., proximity to Nox) and the mitochondria.

Many examples of NP applications still rely on the use of encapsulated or hybridized ROS sensitive fluorescent probes. The caveats associated with fluorescent dye use to sense ROS remain, including the irreversible nature of the oxidation of said dyes, making long term imaging difficult. In addition, the use of DCFH-DA-related dyes as specific ROS sensors has been challenged and recently reviewed in a position paper from a number of leading experts in the Free Radical Field [24]. Problems associated with use of these include lack of specificity, light induced oxidation, and production of ROS [11–17]. The same caution should therefore be taken when using these dyes embedded or conjugated to NPs.

At present, our understanding of the long-term effects of NP and CNT exposure is still in its infancy. Reports from the laboratory generally show little toxicity in cell and animal studies. However, there is an increasing body of evidence that metal NPs in particular have deleterious effects on cells and tissues [102–105]. Importantly, reports point to surges in ROS production, leading to enhanced ROS signaling, inflammation and cytotoxicity [102,105–107]. These effects will obviously depend on the physico-chemical properties of the NP in question, but should nevertheless be considered when using these for intracellular and *in vivo* ROS sensing.

The unique physico-chemical properties of these nanoscale sensors make them attractive targets for potential sensing in the clinical setting. While NPs for drug delivery have been FDA approved for some time, a number of NPs are currently in pre-clinical and clinical trials for combination drug delivery and diagnostic imaging, also referred to as theranostics [108]. Again, their bioavailability, stability and cytotoxicity will have to be carefully investigated and monitored. Using ROS sensing in future clinical imaging will provide us with invaluable data on the patients’ disease progression and response to therapeutic agents.

## Figures and Tables

**Table 1:** NP- and CNT-based ROS sensor characteristics.

Sensor mechanism	Sensitivity & specificity	Intracellular/*in vivo* applications	Advantage	Disadvantage	Studies
**Nanoparticles**					
ROS-dye encapsulation (polymer- & micelle-based)	nM–μM various ROS and RNS, sensitivity limited to dye properties, encapsulation may provide barrier for short-lived ROS	Intracellular: following LPS macrophage stimulation; *in vivo* inflammatory ROS surges associated with tumor xenografts	High sensitivity and strong signal; protection of dye interaction with intracellular molecules, potential for better sub-cellular targeting; embedding of reference dye	Many disadvantages of traditional ROS dyes remain, such as lack of specificity and potential creation of artifacts; irreversible; unstable	[34–40]
Luminescence (polymer- & micelle-based)	nM–μM ROS, H_2_O_2_ Specificity dependent on NP design	Intracellular; *in vivo* intraperitoneal murine inflammatory model	No optical excitation signal; nearinfrared emission allows for tissue imaging; potential future use for ROS induced Photodynamic therapy	Specificity to a particular ROS not evaluated in many studies; stability	[41–45]
Fluorescent-quenching (Metallic)	nM–μM ROS/RNS. Specificity dependent on NP design	Intracellular: following LPS macrophage stimulation, and PDGF treatment	Lack of photo-bleaching; near-infrared spectrum; reversible depending on design; stable; strong fluorescent signal	Potential Intracellular ROS production following metallic NP exposure, potential metallic NP-induced cytotoxicity	[46–50]
NP Surface energy transfer (NSET) (Metallic)	μM range ROS	Intracellular: following LPS macrophage stimulation	stable in high reducing environments	Irreversible	[51]
Surface enhanced Raman Scattering/spectroscopy (SERS) (Metallic shell)	Intracellular redox potential (−394 mV to 370 mM)	Intracellular: redox potential in response to reducing and oxidizing agents.	No optical excitation; reversible; stable	pH sensitive; requires access to Raman microscopy	[52]
**Carbon nanotubes**					
Electrochemical (Oxidase/Peroxidase immobilized; Nanoparticle-doped)	nM–mM H_2_O_2_	Cell-free	Speed of detection, sensitivity	Impractical for whole-cell/*in vivo* applications	[53–60]
Optical (fluorescence quenching)	μM-single molecule H_2_O_2_ detection; NO	Intracellular: in response to VEGF & EGF; *in vivo*: dialysis membrane within IP cavity	Specificity; spatio-temporal resolution; single molecule detection	Slow detection rate; complex mathematical analysis; cell culturing required on CNT arrays	[61–68]

## References

[1] Paulsen C.E., Carroll K.S. (2010). Orchestrating redox signaling networks through regulatory cysteine switches. ACS Chem. Biol.

[2] Tonks N.K. (2005). Redox redux: Revisiting PTPs and the control of cell signaling. Cell.

[3] Tonks N.K. (2006). Protein tyrosine phosphatases: From genes, to function, to disease. Nat. Rev. Mol. Cell Biol.

[4] Forman H.J., Maiorino M., Ursini F. (2010). Signaling functions of reactive oxygen species. Biochemistry.

[5] Lukosz M., Jakob S., Büchner N., Zschauer T.-C., Altschmied J., Haendeler J. (2010). Nuclear redox signaling. Antioxid. Redox Signal.

[6] Starkov A.A. (2008). The role of mitochondria in reactive oxygen species metabolism and signaling. Ann. N. Y. Acad. Sci.

[7] Hamanaka R.B., Chandel N.S. (2010). Mitochondrial reactive oxygen species regulate cellular signaling and dictate biological outcomes. Tr. Biochem. Sci.

[8] Brown D.I., Griendling K.K. (2009). Nox proteins in signal transduction. Free Radic. Biol. Med.

[9] Petry A., Weitnauer M., Görlach A. (2010). Receptor activation of NADPH oxidases. Antioxid. Redox Signal.

[10] Ushio-Fukai M. (2009). Compartmentalization of redox signaling through NADPH oxidase-derived ROS. Antioxid. Redox Signal.

[11] Burkitt M.J., Wardman P. (2001). Cytochrome C is a potent catalyst of dichlorofluorescin oxidation: Implications for the role of reactive oxygen species in apoptosis. Biochem. Biophys. Res. Commun.

[12] Karlsson M., Kurz T., Brunk U.T., Nilsson S.E., Frennesson C.I. (2010). What does the commonly used DCF test for oxidative stress really show?. Biochem. J.

[13] LeBel C.P., Ischiropoulos H., Bondy S.C. (1992). Evaluation of the probe 2′,7′-dichlorofluorescin as an indicator of reactive oxygen species formation and oxidative stress. Chem. Res. Toxicol.

[14] Possel H., Noack H., Augustin W., Keilhoff G., Wolf G. (1997). 2,7-Dihydrodichlorofluorescein diacetate as a fluorescent marker for peroxynitrite formation. FEBS Lett.

[15] Rota C., Chignell C.F., Mason R.P. (1999). Evidence for free radical formation during the oxidation of 2′-7′-dichlorofluorescin to the fluorescent dye 2′-7′-dichlorofluorescein by horseradish peroxidase: Possible implications for oxidative stress measurements. Free Radic. Biol. Med.

[16] Rota C., Fann Y.C., Mason R.P. (1999). Phenoxyl free radical formation during the oxidation of the fluorescent dye 2′,7′-dichlorofluorescein by horseradish peroxidase. Possible consequences for oxidative stress measurements. J. Biol. Chem.

[17] Wrona M., Patel K.B., Wardman P. (2008). The roles of thiol-derived radicals in the use of 2′,7′-dichlorodihydrofluorescein as a probe for oxidative stress. Free Radic. Biol. Med.

[18] Chen X., Tian X., Shin I., Yoon J. (2011). Fluorescent and luminescent probes for detection of reactive oxygen and nitrogen species. Chem. Soc. Rev.

[19] Lippert A.R., Van de Bittner G.C., Chang C.J. (2011). Boronate oxidation as a bioorthogonal reaction approach for studying the chemistry of hydrogen peroxide in living systems. Acc. Chem. Res.

[20] Dickinson B.C., Huynh C., Chang C.J. (2010). A palette of fluorescent probes with varying emission colors for imaging hydrogen peroxide signaling in living cells. J. Am. Chem. Soc.

[21] Dickinson B.C., Srikun D., Chang C.J. (2010). Mitochondrial-targeted fluorescent probes for reactive oxygen species. Curr. Opin. Chem. Biol.

[22] Miller E.W., Albers A.E., Pralle A., Isacoff E.Y., Chang C.J. (2005). Boronate-based fluorescent probes for imaging cellular hydrogen peroxide. J. Am. Chem. Soc.

[23] Kalyanaraman B. (2011). Oxidative chemistry of fluorescent dyes: Implications in the detection of reactive oxygen and nitrogen species. Biochem. Soc. Trans.

[24] Kalyanaraman B., Darley-Usmar V., Davies K.J.A., Dennery P.A., Forman H.J., Grisham M.B., Mann G.E., Moore K., Roberts L.J., Ischiropoulos H. (2012). Measuring reactive oxygen and nitrogen species with fluorescent probes: Challenges and limitations. Free Radic. Biol. Med.

[25] Hilderbrand S.A., Weissleder R. (2010). Near-infrared fluorescence: Application to *in vivo* molecular imaging. Curr. Opin. Chem. Biol.

[26] Karton-Lifshin N., Segal E., Omer L., Portnoy M., Satchi-Fainaro R., Shabat D. (2011). A unique paradigm for a Turn-ON near-infrared cyanine-based probe: Noninvasive intravital optical imaging of hydrogen peroxide. J. Am. Chem. Soc.

[27] Belousov V.V., Fradkov A.F., Lukyanov K.A., Staroverov D.B., Shakhbazov K.S., Terskikh A.V., Lukyanov S. (2006). Genetically encoded fluorescent indicator for intracellular hydrogen peroxide. Nat. Method.

[28] Meyer A.J., Dick T.P. (2010). Fluorescent protein-based redox probes. Antioxid. Redox Signal.

[29] Niethammer P., Grabher C., Look A.T., Mitchison T.J. (2009). A tissue-scale gradient of hydrogen peroxide mediates rapid wound detection in zebrafish. Nature.

[30] Dooley C.T., Dore T.M., Hanson G.T., Jackson W.C., Remington S.J., Tsien R.Y. (2004). Imaging dynamic redox changes in mammalian cells with green fluorescent protein indicators. J. Biol. Chem.

[31] Hanson G.T., Aggeler R., Oglesbee D., Cannon M., Capaldi R.A., Tsien R.Y., Remington S.J. (2004). Investigating mitochondrial redox potential with redox-sensitive green fluorescent protein indicators. J. Biol. Chem.

[32] Wang J., Sui M., Fan W. (2010). Nanoparticles for tumor targeted therapies and their pharmacokinetics. Curr. Drug Metabolism.

[33] Waite C.L., Roth C.M. (2012). Nanoscale drug delivery systems for enhanced drug penetration into solid tumors: Current progress and opportunities. Crit. Rev. Biomed. Eng.

[34] Kim G., Lee Y.E., Xu H., Philbert M.A., Kopelman R. (2010). Nanoencapsulation method for high selectivity sensing of hydrogen peroxide inside live cells. Anal. Chem.

[35] Cao Y., Koo Y.E., Koo S.M., Kopelman R. (2005). Ratiometric singlet oxygen nano-optodes and their use for monitoring photodynamic therapy nanoplatforms. Photochem. Photobiol.

[36] King M., Kopelman M.D. (2003). Development of a hydroxyl radical ratiometric nanoprobe. Sens. Actuator. B Chem.

[37] Tian J., Chen H., Zhuo L., Xie Y., Li N., Tang B. (2011). A highly selective, cell-permeable fluorescent nanoprobe for ratiometric detection and imaging of peroxynitrite in living cells. Chemistry.

[38] Hammond V.J., Aylott J.W., Greenway G.M., Watts P., Webster A., Wiles C. (2008). An optical sensor for reactive oxygen species: Encapsulation of functionalised silica nanoparticles into silicate nanoprobes to reduce fluorophore leaching. Analyst.

[39] Kim S.-H., Kim B., Yadavalli V.K., Pishko M.V. (2005). Encapsulation of enzymes within polymer spheres to create optical nanosensors for oxidative stress. Anal. Chem.

[40] Kim J.Y., Choi W.I., Kim Y.H., Tae G. (2011). Highly selective *in-vivo* imaging of tumor as an inflammation site by ROS detection using hydrocyanine-conjugated, functional nano-carriers. J. Control. Release.

[41] Lee D., Khaja S., Velasquez-Castano J.C., Dasari M., Sun C., Petros J., Taylor W.R., Murthy N. (2007). *In vivo* imaging of hydrogen peroxide with chemiluminescent nanoparticles. Nat. Mater.

[42] Lee D., Erigala V.R., Dasari M., Yu J., Dickson R.M., Murthy N. (2008). Detection of hydrogen peroxide with chemiluminescent micelles. Int. J. Nanomed.

[43] Dasari M., Lee D., Erigala V.R., Murthy N. (2009). Chemiluminescent PEG-PCL micelles for imaging hydrogen peroxide. J. Biomed. Mater. Res. Part A.

[44] Lim C.-K., Lee Y.-D., Na J., Oh J.M., Her S., Kim K., Choi K., Kim S., Kwon I.C. (2010). Chemiluminescence-generating nanoreactor formulation for near-infrared imaging of hydrogen peroxide and glucose level *in vivo*. Adv. Funct. Mater.

[45] Chen R., Zhang L., Gao J., Wu W., Hu Y., Jiang X. (2011). Chemiluminescent nanomicelles for imaging hydrogen peroxide and self-therapy in photodynamic therapy. J. Biomed. Biotechnol.

[46] Wen F., Dong Y., Feng L., Wang S., Zhang S., Zhang X. (2011). Horseradish peroxidase functionalized fluorescent gold nanoclusters for hydrogen peroxide sensing. Anal. Chem.

[47] Shiang Y.-C., Huang C.-C., Chang H.-T. (2009). Gold nanodot-based luminescent sensor for the detection of hydrogen peroxide and glucose. Chem. Commun.

[48] Li D.W., Qin L.X., Li Y., Nia R.P., Long Y.T., Chen H.Y. (2011). CdSe/ZnS quantum dot-Cytochrome c bioconjugates for selective intracellular O2 (−) sensing. Chem. Commun. (Camb).

[49] Wang S., Han M.Y., Huang D. (2009). Nitric oxide switches on the photoluminescence of molecularly engineered quantum dots. J. Am. Chem. Soc.

[50] Casanova D., Bouzigues C., Nguyên T.L., Ramodiharilafy R.O., Bouzhir-Sima L., Gacoin T., Boilot J.P., Tharaux P.L., Alexandrou A. (2009). Single europium-doped nanoparticles measure temporal pattern of reactive oxygen species production inside cells. Nat. Nanotechnol.

[51] Lee H., Lee K., Kim I.-K., Park T.G. (2009). Fluorescent gold nanoprobe sensitive to intracellular reactive oxygen species. Adv. Funct. Mater.

[52] Auchinvole C.A., Richardson P., McGuinnes C., Mallikarjun V., Donaldson K., McNab H., Campbell C.J. (2012). Monitoring intracellular redox potential changes using SERS nanosensors. ACS Nano.

[53] Guo C., Hu F., Li C.M., Shen P.K. (2008). Direct electrochemistry of hemoglobin on carbonized titania nanotubes and its application in a sensitive reagentless hydrogen peroxide biosensor. Biosens. Bioelectron.

[54] Hrapovic S., Liu Y., Keith B., Luong J.H.T. (2004). Electrochemical biosensing platforms using platinum nanoparticles and carbon nanotubes. Anal. Chem.

[55] Yu X., Chattopadhyay D., Galeska I., Papadimitrakopoulos F., Rusling J.F. (2003). Peroxidase activity of enzymes bound to the ends of single-wall carbon nanotube forest electrodes. Electrochem. Commun.

[56] Zeng X., Li X., Liu X., Liu Y., Luo S., Kong B., Yang S., Wei W. (2009). A third-generation hydrogen peroxide biosensor based on horseradish peroxidase immobilized on DNA functionalized carbon nanotubes. Biosens. Bioelectron.

[57] Wang J. (2005). Carbon-nanotube based electrochemical biosensors: A review. Electroanalysis.

[58] Balasubramanian K., Burghard M. (2006). Biosensors based on carbon nanotubes. Anal. Bioanal. Chem.

[59] Besteman K., Lee J.O., Wiertz F.G.M., Heering H.A., Dekker C. (2003). Enzyme-coated carbon nanotubes as single-molecule biosensors. Nano Lett.

[60] Xu J.Z., Zhu J.J., Wu Q., Hu Z., Chen H.Y. (2003). An amperometric biosensor based on the coimmobilization of horseradish peroxidase and methylene blue on a carbon nanotubes modified electrode. Electroanalysis.

[61] Jin H., Heller D.A., Kalbacova M., Kim J.H., Zhang J., Boghossian A.A., Maheshri N., Strano M.S. (2010). Detection of single-molecule H2O2 signalling from epidermal growth factor receptor using fluorescent single-walled carbon nanotubes. Nature Nanotechnol.

[62] Kim J.H., Patra C., Arkalgud J.R., Boghossian A.A., Zhang J., Han J.H., Reuel N.F., Ahn J.H., Mukhopadhyay D., Strano M.S. (2011). Single molecule detection of H_2_O_2_ mediating angiogenic redox signaling on fluorescent single-walled carbon nanotube array. ACS Nano.

[63] Karachevtsev V., Glamazda A.Y., Leontiev V., Lytvyn O., Dettlaff-Weglikowska U. (2007). Glucose sensing based on NIR fluorescence of DNA-wrapped single-walled carbon nanotubes. Chem. Phys. Lett.

[64] Song C., Pehrsson P.E., Zhao W. (2005). Recoverable solution reaction of HiPco carbon nanotubes with hydrogen peroxide. J. Phys. Chem. B.

[65] Song C., Pehrsson P.E., Zhao W. (2006). Optical enzymatic detection of glucose based on hydrogen peroxide-sensitive HiPco carbon nanotubes. J. Mater. Res.

[66] Barone P.W., Baik S., Heller D.A., Strano M.S. (2004). Near-infrared optical sensors based on single-walled carbon nanotubes. Nat. Mater.

[67] Strano M.S., Baik S., Barone P (2007). Sensors Employing Single-Walled Carbon Nanotubes.

[68] Kim J.H., Heller D.A., Jin H., Barone P.W., Song C., Zhang J., Trudel L.J., Wogan G.N., Tannenbaum S.R., Strano M.S. (2009). The rational design of nitric oxide selectivity in single-walled carbon nanotube near-infrared fluorescence sensors for biological detection. Nat. Chem.

[69] Resch-Genger U., Grabolle M., Cavaliere-Jaricot S., Nitschke R., Nann T. (2008). Quantum dots versus organic dyes as fluorescent labels. Nat. Method.

[70] Lee Y.-E.K., Kopelman R. (2009). Optical nanoparticle sensors for quantitative intracellular imaging. Wiley Interdisciplin. Rev. Nanomed. Nanobiotechnol.

[71] Lee Y.-E.K., Smith R., Kopelman R. (2009). Nanoparticle PEBBLE sensors in live cells and *in vivo*. Annu. Rev. Anal. Chem. (Palo Alto Calif).

[72] Albers A.E., Dickinson B.C., Miller E.W., Chang C.J. (2008). A red-emitting naphthofluorescein-based fluorescent probe for selective detection of hydrogen peroxide in living cells. Bioorg. Med. Chem. lett.

[73] Chen W.T., Tung C.H., Weissleder R. (2004). Imaging reactive oxygen species in arthritis. Mol. Imag.

[74] Devalapally H., Shenoy D., Little S., Langer R., Amiji M. (2007). Poly(ethylene oxide)-modified poly(beta-amino ester) nanoparticles as a pH-sensitive system for tumor-targeted delivery of hydrophobic drugs: Part 3. Therapeutic efficacy and safety studies in ovarian cancer xenograft model. Cancer Chemother. Pharmacol.

[75] Bruchez M., Moronne M., Gin P., Weiss S., Alivisatos A.P. (1998). Semiconductor nanocrystals as fluorescent biological labels. Science.

[76] Elghanian R., Storhoff J.J., Mucic R.C., Letsinger R.L., Mirkin C.A. (1997). Selective colorimetric detection of polynucleotides based on the distance-dependent optical properties of gold nanoparticles. Science.

[77] Michalet X., Pinaud F.F., Bentolila L.A., Tsay J.M., Doose S., Li J.J., Sundaresan G., Wu A.M., Gambhir S.S., Weiss S. (2005). Quantum dots for live cells, *in vivo* imaging, and diagnostics. Science.

[78] Wu Z., Jin R. (2010). On the ligand’s role in the fluorescence of gold nanoclusters. Nano Lett.

[79] Aguila A., Murray R.W. (2000). Monolayer-protected clusters with fluorescent dansyl ligands. Langmuir.

[80] Demers L.M., Mirkin C.A., Mucic R.C., Reynolds R.A., Letsinger R.L., Elghanian R., Viswanadham G. (2000). A fluorescence-based method for determining the surface coverage and hybridization efficiency of thiol-capped oligonucleotides bound to gold thin films and nanoparticles. Anal. Chem.

[81] Dulkeith E., Ringler M., Klar T.A., Feldmann J., Munoz Javier A., Parak W.J. (2005). Gold nanoparticles quench fluorescence by phase induced radiative rate suppression. Nano Lett.

[82] Yun C.S., Javier A., Jennings T., Fisher M., Hira S., Peterson S., Hopkins B., Reich N.O., Strouse G.F. (2005). Nanometal surface energy transfer in optical rulers, breaking the FRET barrier. J. Am. Chem. Soc.

[83] Guarise C., Pasquato L., De Filippis V., Scrimin P. (2006). Gold nanoparticles-based protease assay. Proc. Natl. Acad. Sci. USA.

[84] Kneipp J., Kneipp H., Wittig B., Kneipp K. (2010). Novel optical nanosensors for probing and imaging live cells. Nanomed-Nanotechnol.

[85] Strano M.S., Boghossian A.A., Kim W.J., Barone P.W., Jeng E.S., Heller D.A., Nair N., Jin H., Sharma R., Lee C.Y. (2009). The Chemistry of Single-Walled Nanotubes. MRS Bull.

[86] Cheung W., He H, Li S., Singh J., Banerjee I.A. (2011). Carbon Nanotubes: *In Vitro* and *in Vivo* Sensing and Imaging. Biosensor Nanomaterials.

[87] Dai H. (2002). Carbon nanotubes: Opportunities and challenges. Surf. Sci.

[88] O’Connell M.J., Bachilo S.M., Huffman C.B., Moore V.C., Strano M.S., Haroz E.H., Rialon K.L., Boul P.J., Noon W.H., Kittrell C. (2002). Band gap fluorescence from individual single-walled carbon nanotubes. Science.

[89] Strano M.S., Huffman C.B., Moore V.C., O’Connell M.J., Haroz E.H., Hubbard J., Miller M., Rialon K., Kittrell C., Ramesh S. (2003). Reversible, band-gap-selective protonation of single-walled carbon nanotubes in solution. J. Phys. Chem. B.

[90] Wang J., Musameh M., Lin Y. (2003). Solubilization of carbon nanotubes by nafion toward the preparation of amperometric biosensors. J. Am. Chem. Soc.

[91] Lee C.-S., Baker S.E., Marcus M.S., Yang W., Eriksson M.A., Hamers R.J. (2004). Electrically addressable biomolecular functionalization of carbon nanotube and carbon nanofiber electrodes. Nano Lett.

[92] Peng H., Alemany L.B., Margrave J.L., Khabashesku V.N. (2003). Sidewall carboxylic acid functionalization of single-walled carbon nanotubes. J. Am. Chem. Soc.

[93] Chen R.J., Bangsaruntip S., Drouvalakis K.A., Kam N.W.S., Shim M., Li Y., Kim W., Utz P.J., Dai H. (2003). Noncovalent functionalization of carbon nanotubes for highly specific electronic biosensors. Proc. Natl. Acad. Sci.

[94] Liu C.Y., Hu J.M. (2009). Hydrogen peroxide biosensor based on the direct electrochemistry of myoglobin immobilized on silver nanoparticles doped carbon nanotubes film. Biosens. Bioelectron.

[95] Cognet L., Tsyboulski D.A., Rocha J.D.R., Doyle C.D., Tour J.M., Weisman R.B. (2007). Stepwise quenching of exciton fluorescence in carbon nanotubes by single-molecule reactions. Science.

[96] Satishkumar B., Brown L.O., Gao Y., Wang C.C., Wang H.L., Doorn S.K. (2007). Reversible fluorescence quenching in carbon nanotubes for biomolecular sensing. Nat. Nanotechnol.

[97] Barone P.W., Baik S., Heller D.A., Strano M.S. (2005). Modulating single walled carbon nanotube fluorescence in response to specific molecular adsorption. AIP Conf. Proc.

[98] Heller D.A., Jin H., Martinez B.M., Patel D., Miller B.M., Yeung T.K., Jena P.V., Höbartner C., Ha T., Silverman S.K. (2009). Multimodal optical sensing and analyte specificity using single-walled carbon nanotubes. Nat. Nanotechnol.

[99] Xu Y., Pehrsson P.E., Chen L., Zhang R., Zhao W. (2007). Double-stranded DNA single-walled carbon nanotube hybrids for optical hydrogen peroxide and glucose sensing. J. Phys. Chem. C.

[100] Patra C.R., Kim J.H., Pramanik K., d’Uscio L.V., Patra S., Pal K., Ramchandran R., Strano M.S., Mukhopadhyay D. (2011). Reactive oxygen species driven angiogenesis by inorganic nanorods. Nano Lett.

[102] Marano F., Hussain S., Rodrigues-Lima F., Baeza-Squiban A., Boland S. (2011). Nanoparticles: Molecular targets and cell signalling. Arch. Toxicol.

[101] Zhang J., Boghossian A.A., Barone P.W., Rwei A., Kim J.H., Lin D., Heller D.A., Hilmer A.J., Nair N., Reuel N.F. (2011). Single molecule detection of nitric oxide enabled by d (AT) 15 DNA adsorbed to near infrared fluorescent single-walled carbon nanotubes. J. Am. Chem. Soc.

[103] Nel A., Xia T., Madler L., Li N. (2006). Toxic potential of materials at the nanolevel. Science.

[104] Nel A.E., Madler L., Velegol D., Xia T., Hoek E.M., Somasundaran P., Klaessig F., Castranova V., Thompson M. (2009). Understanding biophysicochemical interactions at the nano-bio interface. Nat. Mater.

[105] Zhao F., Zhao Y., Liu Y., Chang X., Chen C., Zhao Y. (2011). Cellular uptake, intracellular trafficking, and cytotoxicity of nanomaterials. Small.

[106] Pulskamp K., Diabaté S., Krug H.F. (2007). Carbon nanotubes show no sign of acute toxicity but induce intracellular reactive oxygen species in dependence on contaminants. Toxicol. Lett.

[107] Sharma C.S., Sarkar S., Periyakaruppan A., Barr J., Wise K., Thomas R., Wilson B.L., Ramesn G.T. (2007). Single-walled carbon nanotubes induces oxidative stress in rat lung epithelial cells. J. Nanosci. Nanotechnol.

[108] Jokerst J.V., Gambhir S.S. (2011). Molecular imaging with theranostic nanoparticles. Acc. Chem. Res.

